# *Euonymus alatus* (Thunb.) Siebold Prevents Osteoclast Differentiation and Osteoporosis

**DOI:** 10.3390/nu15183996

**Published:** 2023-09-15

**Authors:** Sung-Ju Lee, Seon-A Jang, Seong Cheol Kim, Dong Ryun Gu, Hyun Yang, Jin Ah Ryuk, Hyunil Ha

**Affiliations:** 1 KM Convergence Research Division, Korea Institute of Oriental Medicine, Yuseong-daero 1672, Daejeon 34054, Republic of Korea; sungjulee@kiom.re.kr (S.-J.L.); iron0907@kiom.re.kr (S.C.K.); mrwonsin@kiom.re.kr (D.R.G.); hyunyang@kiom.re.kr (H.Y.); yukjinah@kiom.re.kr (J.A.R.); 2 Future Technology Research Center, KT&G Corporation, 30, Gajeong-ro, Yuseong-gu, Daejeon 34128, Republic of Korea; jsa85@ktng.com

**Keywords:** *Euonymus alatus* (Thunb.) Siebold, osteoporosis, bone loss, ovariectomy

## Abstract

*Euonymus alatus* (Thunb.) Siebold, a traditional medicinal plant, has been used in China and several other Asian countries to address a variety of health concerns. The extensive research conducted on *E. alatus* is driven by its diverse pharmacological applications. However, its biological effects on osteoclastogenesis and osteoporosis have not been previously studied. In this research, we investigated the impact of an ethanolic extract of *E. alatus* (EEEA) on osteoclast differentiation and function as well as estrogen deficiency-induced bone loss. We found that EEEA inhibits osteoclast differentiation by downregulating the expression of the receptor activator of nuclear factor-κB ligand (RANKL) in osteoclast-supporting cells and by directly impeding RANKL-mediated signaling pathways for osteoclastogenesis in precursor cells. In addition, EEEA inhibited the bone-resorptive function of mature osteoclasts in vitro. Furthermore, oral administration of EEEA significantly alleviated bone loss in an ovariectomy-induced osteoporosis mouse model. Additionally, we identified phytochemicals in EEEA that have suppressive effects on osteoclast differentiation and bone loss. Collectively, these results suggest that EEEA holds potential as a biotherapeutic candidate for anti-postmenopausal osteoporosis.

## 1. Introduction

Bone is a living, dynamic tissue that constantly renews through a tightly connected process of osteoblastic bone formation and osteoclastic bone resorption [1]. Osteolytic bone diseases, including postmenopausal osteoporosis, occur due to elevated osteoclast formation or excessive activation of osteoclast activity [2]. Therefore, osteoclasts are the primary targets for the development of new therapeutic agents for anti-osteoporosis treatment [2,3]. Pharmacological treatments for osteoporosis encompass a range of therapeutic agents, such as nitrogen-containing bisphosphonates, denosumab (the anti-receptor activator of nuclear factor-κB ligand (RANKL) antibody), and romosozumab (anti-sclerostin antibody) [3,4,5,6]. Although these agents effectively alleviate bone loss, they can also cause critical side effects such as vomiting, stomach pain, renal and gastrointestinal toxicities, endometrial cancer, breast cancer, and myocardial infarction when used long-term [7]. Therefore, there is a desperate need for natural products such as herbal medicines and foods that can serve as prophylactic or therapeutic agents for osteoporosis and other osteolytic bone diseases, with low toxicity and fewer side effects [8,9]. Osteoclasts are multinucleated giant cells derived from the monocyte/macrophage lineage in response to two vital cytokines, RANKL and macrophage-colony stimulating factor (M-CSF) [2]. M-CSF plays an important role in osteoclast precursor proliferation and survival [10]. RANKL plays a pivotal role in the process of osteoclast development by inducing and activating the key transcription factors, namely the nuclear factor of activated T cells c1 (NFATc1) and cellular oncogene fos (c-Fos), through the downstream signaling pathways of RANKL’s receptor, including nuclear factor-κB (NF-κB) and mitogen-activated protein kinases (MAPKs) such as c-Jun N-terminal protein kinase (JNK) and p38 [10,11]. NFATc1, regarded as the central transcriptional regulator, governs the expression of a multitude of osteoclast-specific genes, including cathepsin K (Ctsk), tartrate-resistant acid phosphatase (TRAP), dendritic cell-specific transmembrane proteins (DC-stamp), and ATPase H+ transporting V0 subunit D2 (Atp6v0d2) [11,12].

Following their differentiation, mature osteoclasts require activation in order to initiate the degradation of the bone matrix. These multinucleated osteoclasts undergo polarization towards the bone surface, facilitated by cytoskeletal rearrangement, and subsequently establish a tightly sealed resorption compartment positioned between the bone surface and the osteoclasts. Within this sealed compartment, osteoclasts secrete acids and lytic enzymes to facilitate the degradation of both bone mineral and the organic matrix. It is noteworthy that this osteoclast activation process is modulated by RANKL as well [13]. Given these insights, the suppression of osteoclast differentiation and the activation pathways regulated by RANKL present a promising strategy for the mitigation and control of bone loss associated with osteoclast activity, particularly in conditions such as postmenopausal osteoporosis.

*Euonymus alatus* (Thunb.) Siebold has a long medicinal history of use as a traditional medicinal plant in Asia. The medicinal efficacy of *E. alatus* was first recorded in the earliest Chinese medical monograph, the ShenNong BenCaoJing, and was discussed in the traditional Chinese medicine monographs of later dynasties. Moreover, the cork cambium of its twigs and wings, known as ‘Gui-jun-woo’, has historically been attributed to various functions, including enhancing blood circulation, alleviating pain, eliminating stagnant blood, and treating dysmenorrhea in past dynasties. [14,15,16,17,18,19]. In the RiHuaZi BenCao, another well-known traditional Chinese medicine (TCM) classic, *E. alatus* was recorded as a drug with the function of modulating gynecological diseases [19]. Furthermore, recent studies have substantiated the effects of *E. alatus*, including its efficacy in treating rheumatoid arthritis, its immune-modulating characteristics, its benefits in managing hyperglycemia and diabetic retinopathy, along with its anti-inflammatory attributes [14,15,16,17,19,20]. Despite these findings, the potential impact of *E. alatus* on bone metabolism and bone loss remains uninvestigated. Therefore, this study aimed to explore the inhibitory effects of an ethanolic extract of *E. alatus* (EEEA) on osteoclast differentiation and function, as well as its potential bone-protective effect, using an ovariectomy (OVX)-induced bone loss mouse model.

## 2. Materials and Methods

### 2.1. Materials

MLO-Y4 cells were obtained from Kerafast (Boston, MA, USA). Primary antibodies against phospho-IκBα (Ser32) (#2859), phospho-p38 (#9211), phospho-SAPK/JNK (Thr183/Tyr185) (#9251), phospho-p44/42 MAPK (Erk1/2) (Thr202/Tyr204) (#9101), IκBα (#9242), p38 (#9212), SAPK/JNK (#9252), p44/42 MAPK (Erk1/2) (#9102), and β-Actin (#3700) were obtained from Cell Signaling Technology (Danvers, MA, USA). Anti-rabbit and anti-mouse HRP-conjugated secondary antibodies were purchased from Santa Cruz Biotechnology (Santa Cruz, CA, USA). The RNA-spinTM extraction kit was purchased from iNtRON Biotechnology (Seongnam, Republic of Korea), and the High-Capacity cDNA Reverse Transcription Kit was obtained from Thermo Fisher Scientific (Waltham, MA, USA). A TaqMan Universal Master Mix II was obtained from Applied Biosystems (Foster City, CA, USA). Dried cork cambiums of *E. alatus* were bought from Omniherb (Yeongcheon, Republic of Korea). They were extracted using 70% ethanol in a 15-fold volume, under reflux conditions at 80 °C for 3 h. After filtration, the extract was then lyophilized. The EEEA powder was dissolved in dimethyl sulfoxide for the in vitro experiments or in distilled water for the animal experiments.

### 2.2. Preparation of Osteoclast Precursor Cells

Freshly flushed bone marrow macrophages (BMMs) were obtained from the femurs and tibias of C57BL/6J mice. The BMMs were cultured in α-MEM supplemented with M-CSF (60 ng/mL) in a cell culture plate for 24 h. Non-adherent cells were harvested and cultured in non-coated plates in α-MEM supplemented with M-CSF (60 ng/mL) for 5 days. The adherent cells, excluding the non-adherent cells in the non-coated plates, were used as BMMs for subsequent experiments, serving as osteoclast precursor cells.

### 2.3. Cell Viability Assay

The BMMs were seeded into 96-well plates at a density of 2 × 10^4^ cells per well and cultured in the presence of M-CSF (60 ng/mL) with or without EEEA (11.1, 33.3, and 100 μg/mL). The BMMs were then incubated with different concentrations of EEEA for 24 h. After that, the CCK-8 reagent was added to each well and incubated for 2 h. The absorbance of the plate was read using a microplate reader (Molecular Devices, San Jose, CA, USA) at 450 nm.

### 2.4. In Vitro Osteoclastogenesis Assay

For the co-culture of the BMMs and MLO-Y4 cells to induce osteoclast differentiation, the MLO-Y4 cells and BMMs were seeded in 96-well plates at densities of 4 × 10^3^ and 4 × 10^4^ cells/well, respectively. On the following day, the BMM-MLO-Y4 co-culture was exposed to 10 nM of 1α,25-dihydroxyvitamin D3 (VitD3) with or without varying concentrations of EEEA (11.1, 33.3, and 100 μg/mL) for a duration of 5 days. In order to explore the direct impact of EEEA on osteoclast differentiation from precursor cells, the BMMs (1 × 10^4^ cells/well) were seeded in 96-well plates and cultured with or without EEEA in the presence of M-CSF (60 ng/mL) and RANKL (50 ng/mL) for a duration of 4 days. The evaluation of osteoclast differentiation was conducted through the measurement of the total cellular TRAP activity and TRAP staining, as described previously [21].

### 2.5. In Vitro Osteoclast Function Assay

To initiate osteoclast differentiation, the BMMs were cultured in a 10 cm culture dish and treated with M-CSF (60 ng/mL) and RANKL (50 ng/mL) for a duration of 2 days. The committed cells were subsequently cultured for an additional 2 days on a culture dish coated with a collagen gel to promote the maturation of osteoclasts. The mature osteoclasts obtained were then seeded onto a Corning Osteo Assay Surface plate (Corning Inc., Corning, NY, USA) using 0.2% collagenase (Sigma-Aldrich, St. Louis, MO, USA). Following a 3 h settlement period, the mature osteoclasts were subjected to treatment with or without EEEA (11.1, 33.3, and 100 μg/mL) for 1 day in the presence of M-CSF and RANKL. Subsequent to cell removal, the resorbed pits were photographed, and the resorbed area was quantified using ImageJ software.

### 2.6. Western Blotting

The cells were harvested and lysed on ice. After the cell lysis, the protein concentrations were measured using a BCA protein assay kit. The protein samples were separated via SDS-polyacrylamide gel electrophoresis and transferred to polyvinylidene difluoride membranes. The membranes were blocked with 5% skim milk for 1 h at room temperature and then incubated with specific primary antibodies overnight at 4 °C. After three washes with Tris-buffered saline plus 0.1% Tween 20, the membranes were incubated with HRP-conjugated secondary antibodies for 1 h. Finally, the proteins were visualized via chemiluminescence using a ChemiDoc Imaging System (Bio-Rad, Hercules, CA, USA).

### 2.7. Quantitative Real-Time Polymerase Chain Reaction (PCR)

Total RNA was extracted using the RNA-spinTM extraction kit, followed by reverse transcription into cDNA using the High-Capacity cDNA Reverse Transcription Kit for the real-time PCR detection of the related gene expression. The synthesized cDNA was amplified using TaqMan Universal Master Mix II and TaqMan probes for the target genes: tumor necrosis factor ligand superfamily member 11 (Tnfsf11, Mm00441908_m1), tumor necrosis factor receptor superfamily member 11B (Tnfrsf11b, Mm0043542_m1), colony stimulating factor 1 (Csf1, Mm00432686_m1), c-Fos (Mm00487425_m1), Nfatc1 (Mm00479445_m1), Atp6v0d2 (Mm00656638_m1), Dc-stamp (Mm01168058_m1), Ctsk (Mm00484036_m1), positive regulatory domain zinc finger protein 1 (Prdm1; encoding Blimp1, Mm00476128_m1), interferon regulatory factor 8 (Irf8, Mm00492567_m1), v-maf avian musculoaponeurotic fibrosarcoma oncogene homolog B (Mafb, Mm00627481_s1), and 18S rRNA (Hs99999901_s1). The amplification was performed using an ABI 7500 Real-Time PCR Instrument (Applied Biosystems).

### 2.8. OVX-Induced Osteoporosis Model

Seven-week-old female C57BL/6J mice were obtained from Japan SLC Inc. (Shizuoka, Japan) and housed under standard conditions with free access to a standard chow diet and water. After one week of acclimatization, the mice underwent either a sham or OVX operation after being anesthetized. Previous studies have demonstrated that the oral administration of *E. alatus* extracts at doses ranging from 50 to 300 mg/kg/day resulted in neuroprotective and immunostimulatory effects in animal models [22,23,24]. Based on these findings, we established an EEEA oral dosage regimen, with 100 mg/kg/day as the low dose and 300 mg/kg/day as the high dose, to assess the impact of EEEA in an OVX-induced mouse bone loss model. The mice were divided into four groups (n = 7 mice per group): sham, OVX, OVX + low-dose EEEA (EEEA-L, 100 mg/kg/day), and high-dose EEEA (EEEA-H, 300 mg/kg/day). Starting one week after the operation, the mice were fed a purified low-fat diet (D12450B, Research Diet, New Brunswick, NJ, USA) and orally administered either distilled water (sham and OVX groups) or EEEA (low-and high-dose groups) every day for six weeks. After the treatment period, the mice were sacrificed, and their femurs were obtained for bone microstructure analysis.

### 2.9. Micro-Computed Tomography (Micro-CT) Analysis

For the structural analysis of the trabecular bone, the distal end of the right femur was scanned using the SkyScan 1276 micro-computed tomography system (Bruker, Kontich, Belgium). The morphometric parameters of the trabecular bone, including the trabecular bone mineral density (BMD; g/cm^3^), bone volume to total volume (BV/TV), trabecular number (Tb. N), trabecular separation (Tb. Sp), and trabecular thickness (Tb. Th), were measured using SkyScan software (version 1.7.42, Bruker, Kontich, Belgium).

### 2.10. Ultrahigh Performance Liquid Chromatography–Tandem Mass Spectrometry (UHPLC-MS/MS) Analysis

The EEEA constituents were analyzed using a Thermo Dionex UltiMate 3000 HPLC system (Dionex Corp., Sunnyvale, CA, USA) coupled with a Thermo Q-Exactive mass spectrometer (Thermo Fisher Scientific, Bremen, Germany). Chromatographic separation was carried out on an Acquity BEH C18 column (1.7 µm, 100 × 2.1 mm). The analysis protocol was based on a previous study with slight modifications [25,26]. The UHPLC-MS/MS analysis targeted the identification of various compounds in EEEA. Two triterpenoids, namely betulinic acid and oleanonic acid, and nine flavonoids, namely catechin, epicatechin, quercetin-3-O-sophoroside, quercetin-3-O-sambubioside, isoquercitrin, kaempferitrin, quercitrin, dihydrokaempferol, and naringenin, were investigated [14,27,28,29,30]. Except for isoquercitrin, these compounds were obtained from ChemFace (Wuhan, China).

### 2.11. Statistical Analysis

Data were indicated as mean ± standard deviation (SD) for the in vitro experiments and as mean ± standard error of the mean (SEM) for the in vivo experiments. Statistical data analysis was performed using a one-way analysis of variance (ANOVA) followed by Dunnett’s test, or two-way ANOVA followed by Sidak’s test. Levels of *p* values below 0.05 were considered statistically significant.

## 3. Results and Discussion

### 3.1. EEEA Inhibits Osteoclast Differentiation

**Figure 1:**
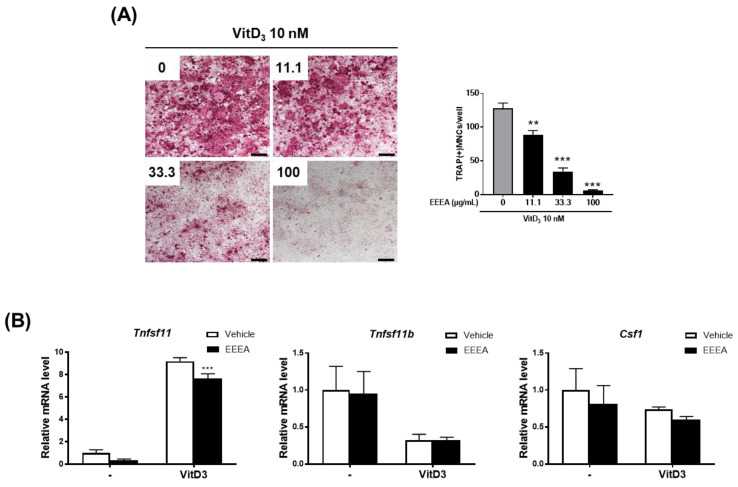
EEEA inhibits osteoclast differentiation in the co-culture of osteoclast precursor cells and osteocyte-like cells. BMMs and MLOY-4 cells were co-cultured for 5 days in the presence of VitD3 (10 nM) with either vehicle (DMSO) or EEEA (11.1, 33.3, and 100 μg/mL). (**A**) Representative microscope images of TRAP staining (left panel; scale bar, 100 µm) and the number of osteoclasts, as well as TRAP-positive multinucleated cells (MNCs) with more than three nuclei (right panel). (**B**) MLO-Y4 cells were treated with or without EEEA (100 μg/mL) and VitD3 (10 nM) for 1 day. mRNA expression levels were evaluated via real-time PCR. ** *p*  <  0.01; *** *p*  <  0.001 versus vehicle control.

**Figure 2:**
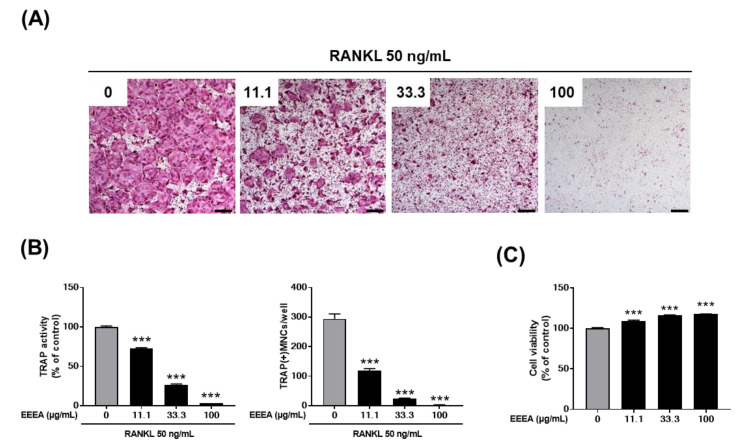
EEEA inhibits RANKL-induced osteoclast differentiation in BMMs. BMMs were cultured for 4 days in the presence of M-CSF (60 ng/mL) and RANKL (50 ng/mL) with either vehicle (DMSO) or EEEA (11.1, 33.3, and 100 μg/mL). (**A**) Representative microscope images of TRAP staining (scale bar, 100 µm). (**B**) Total TRAP activity (left panel) and the number of osteoclasts (right panel). (**C**) Cell viability was determined using the CCK-8 assay. *** *p*  <  0.001 versus vehicle control.

We investigated the effect of EEEA on osteoclast formation in a co-culture of osteocyte-like cells, MLO-Y4, with osteoclast precursor cells, BMMs. Osteocytes are the major cells that produce crucial pro-osteoclastogenic molecules, such as RANKL and its inhibitor osteoprotegerin (OPG), which regulate osteoclast formation and activation [31,32,33]. As shown in Figure 1A, stimulation with VitD3 in the co-culture system for 5 days increased osteoclast formation, which was inhibited by EEEA in a dose-dependent manner. In the co-culture system, the presence of VitD3 promotes osteoclast differentiation from its precursors by upregulating RANKL expression and downregulating OPG expression in MLO-Y4 cells. Therefore, we next examined the impact of EEEA on the mRNA expression of Tnfsf11 (the gene encoding RNAKL), Tnfrsf11b (the gene encoding OPG), and Csf1 (the gene encoding M-CSF) in MLO-Y4 cells. EEEA suppressed the VitD3-induced mRNA expression of Ttnfsf11, whereas it had no effect on Tnfsf11b and Csf1 expression in the basal and VitD3-stimulated conditions (Figure 1B). These results suggest that EEEA could inhibit osteoclast formation by suppressing RANKL expression in the osteoclast-supporting cells, MLO-Y4 cells. We next explored whether EEEA could inhibit osteoclast formation independently of its effect on Ttnfsf11 expression. To verify the direct effect of EEEA on osteoclast differentiation from its precursors, BMM cells were treated with various concentrations of EEEA (0, 11.1, 33.3, and 100 μg/mL) for 5 days in the presence of M-CSF (50 ng/mL) and RANKL (50 ng/mL). According to TRAP activity and staining, RANKL stimulation significantly induced osteoclast formation, and this induction was dose-dependently inhibited by the EEEA treatment (Figure 2A,B). To ensure that EEEA inhibits osteoclast differentiation without causing cytotoxicity, we investigated the cell viability of the BMMs. EEEA did not exhibit any cytotoxic effects on the BMMs but slightly increased cell viability (Figure 2C). These results provide evidence that EEEA directly impedes the osteoclast differentiation process by inhibiting RANKL’s action on osteoclast precursor cells.

### 3.2. EEEA Inhibits RANKL-Induced Signaling Pathways

**Figure 3:**
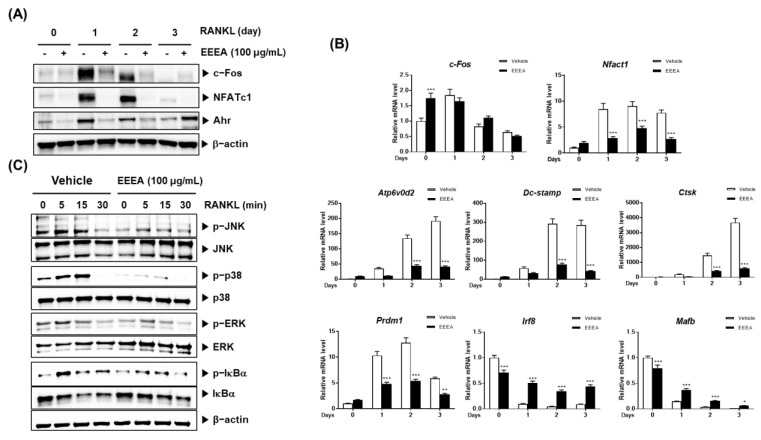
EEEA inhibits RANKL-induced osteoclastogenic transcription factors and osteoclast specific marker genes. BMMs were pretreated with vehicle (DMSO) or EEEA (100 μg/mL) for 3 h and then stimulated with RANKL (50 ng/mL) for the indicated times. (**A**) c-Fos, NFATc1, and Ahr protein levels were evaluated via Western blotting. (**B**) mRNA expression levels were evaluated via real-time PCR. (**C**) The activation of MAPKs and NF-κB pathways was evaluated via Western blotting. * *p*  <  0.05; ** *p*  <  0.01; *** *p*  <  0.001 versus the control.

To explore the mechanism of action by which EEEA exerts its inhibitory role on the differentiation of osteoclast precursors, we examined the effect of EEEA on key transcription factors involved in the RANK signaling pathways for osteoclastogenesis. Among the TNF superfamily members, RANKL binds to the its receptors of osteoclast progenitor cells and plays an important role in promoting osteoclastogenesis [10,34,35]. The RANK-RANKL interaction activates MAPK and NF-κB signaling pathways by recruiting TNF receptor-associated factors (TRAFs). These factors, in turn, induce the activation of c-Fos and NFATc1, which are crucial master modulators for osteoclast differentiation [10,11,35]. These transcription factors positively regulate the expression of genes specific to osteoclasts, including TRAP, ATP6v0d2, CtsK, and Dc-stamp [11,12,35,36]. Upon stimulation with RANKL, both the NFATc1 mRNA and protein expression were elevated, but this increase was significantly inhibited by the EEEA treatment (Figure 3A,B). Correspondingly, the EEEA treatment suppressed RANKL-induced mRNA expression levels of ATP6v0d2, Dc-stamp, and CtsK (Figure 3B). RANKL attenuates the expression of MafB, which acts as a negative osteoclast transcription factor by downregulating the transcriptional activity of NFATc1 [37]. Similarly, IRF-8 has been known as a negative modulator of NFATc1. These negative modulators are negatively regulated by transcriptional suppressors, such as Prdm1 [37,38]. As shown in Figure 3B, the decrease in mRNA levels of IRF-8 and MafB, and the increase in mRNA levels of Prdm1, were suppressed by the EEEA treatment.

The aryl hydrocarbon receptor (AhR) has been shown to play an important role in osteoclast differentiation through its involvement in the RANK/c-Fos axis. Its expression increases during osteoclast differentiation, and the BMMs from the AhR knockout mice exhibit an impaired RANKL-induced activation of MAPKs and NF-κB, as well as c-Fos induction [39,40]. We observed that EEEA inhibits the RANKL-induced protein expression of c-Fos but does not affect its mRNA expression (Figure 3A,B). Additionally, EEEA attenuated the RANKL-induced AhR protein expression (Figure 3A). Early RANK signaling for osteoclast differentiation involves the NF-κB and MAPK signaling pathways [10,38]. In line with the reduction in AhR expression, EEEA significantly inhibited the RANKL-induced activation of MAPKs (ERK, JNK, and p38 phosphorylation) and the classical NF-κB pathway, as demonstrated by the IκBα phosphorylation and degradation (Figure 3C). Our collective findings suggest that EEEA inhibits RANKL-induced osteoclast differentiation by negatively regulating the positive AhR/c-Fos/NFATc1 axis, as well as the negative regulators, IRF-8 and MafB.

### 3.3. EEEA Inhibits the Function of Mature Osteoclasts

**Figure 4:**
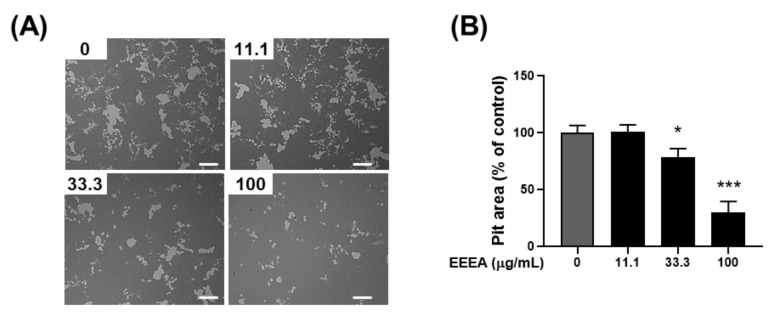
EEEA inhibits the bone-resorptive function of mature osteoclasts. Mature osteoclasts were cultured on Osteo Assay Surface plates in the presence of either vehicle (DMSO) or EEEA (11.1, 33.3, and 100 μg/mL) for 1 day. (**A**) Representative images of resorption pits after cell removal (scale bar, 200 µm). (**B**) Relative quantification of the resorbed area. * *p*  <  0.05; *** *p*  <  0.001 versus vehicle control.

Following the confirmation of EEEA’s efficacy in inhibiting osteoclast differentiation, our investigation extended to its impact on the bone-resorptive function of mature osteoclasts. Mature osteoclasts, as part of their activity, degrade bone mineral through the secretion of acids into a sealed resorption compartment. In this context, we assessed the influence of EEEA on the mineral lytic activity of mature osteoclasts using Osteo Assay Surface plates with a bone-mimetic synthetic surface. After culturing mature osteoclasts on these plates for 1 day, the formation of the resorption pits was observed. Importantly, this formation was dose-dependently inhibited by the EEEA treatment (Figure 4A,B), indicating the capacity of EEEA to impede osteoclast resorptive activity.

### 3.4. EEEA Prevents Bone Loss in OVX Mice

**Figure 5:**
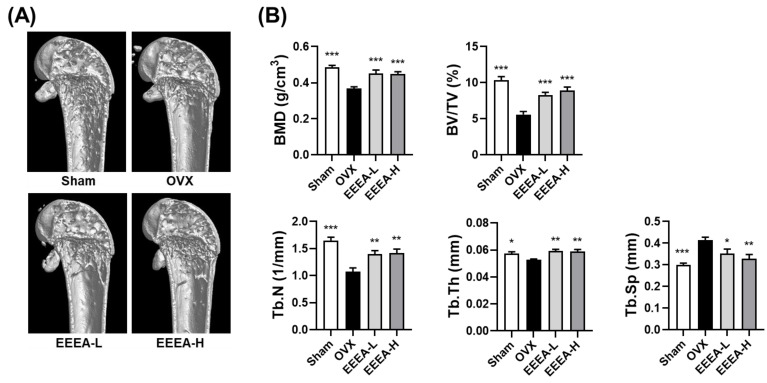
EEEA suppresses the bone loss induced by OVX in vivo. (**A**) Three-dimensional reconstructed images of the distal femurs from each treatment group were obtained using micro-CT. (**B**) Morphometric analysis of distal femoral metaphyseal trabecular bone. Trabecular bone mineral density (BMD), bone volume to total volume (BV/TV), trabecular number (Tb. N), trabecular thickness (Tb. Th), and trabecular separation (Tb. Sp). Data are presented as mean ± standard error of the mean (SEM) for each group, with n = 7. * *p*  <  0.05; ** *p*  <  0.01; *** *p*  <  0.001 versus OVX group.

After discovering the inhibitory effects of EEEA on osteoclast differentiation and function, we investigated the effects of EEEA on bone loss in vivo, utilizing an OVX-induced mouse model of postmenopausal osteoporosis. In this study, one week after the OVX or sham operation, the mice were fed a purified low-fat diet and orally administered EEEA for 6 weeks. As expected, the OVX mice exhibited a significant decrease in trabecular BMD in the distal femur, along with metaphyseal trabecular bone loss. Micro-CT analysis demonstrated that EEEA administration in the OVX mice significantly attenuated bone loss, as evidenced by the increased BMD, BV/TV, Tb.N, and Tb.Th, with decreased trabecular separation (Tb.Sp) compared to the OVX control group (Figure 5A,B). Estrogen is a fundamental hormonal regulator in skeletal growth and bone remodeling for both men and women [41,42]. Postmenopausal osteoporosis is a disease characterized by excessive bone destruction, which results from estrogen deficiency [41,42]. Hormone replacement therapy with estrogen may help prevent bone loss in postmenopausal osteoporosis [41,42]. However, the long-term administration of estrogen may lead to critical side effects such as gastrointestinal toxicities, endometrial cancer, breast cancer, and myocardial infarction [7,41,42]. Therefore, exploring nonpharmaceutical alternatives that leverage the properties of natural products, including herbal medicines and foods, may present a promising avenue for addressing osteoporosis prevention and treatment [8,9].

Our findings highlight the potential of EEEA as a valuable alternative for addressing postmenopausal osteoporosis, given its inhibitory effect on bone loss in the OVX-induced model. However, further research is imperative to elucidate the exact mechanisms responsible for EEEA’s bone-protective effects, particularly its direct impact on osteoclast differentiation and function in vivo.

### 3.5. Phytochemical Constituents of EEEA

**Figure 6:**
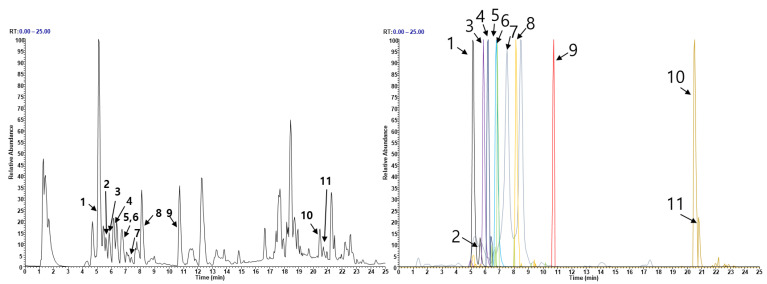
Analysis of EEEA constituents using UHPLC-MS/MS. (**A**) Base peak chromatogram in negative ion mode. (**B**) Extracted ion chromatogram of identified phytochemicals.

To investigate the phytochemical constituents responsible for the anti-osteoporotic effects of EEEA, we conducted a chemical profile analysis using UHPLC-MS/MS. Mass spectra and retention times allowed us to identify the following compounds present in EEEA: two triterpenoids (betulinic acid and oleanonic acid) and nine flavonoids (catechin, epicatechin, quercetin-3-O-sophoroside, quercetin-3-O-sambubioside, isoquercitrin, kaempferitrin, quercitrin, dihydrokaempferol, and naringenin) (Figure 6 and Table 1). Previous studies have reported that the two triterpenoids, betulinic acid and oleanolic acid, can prevent bone loss in ovariectomized mice by inhibiting RANKL-associated osteoclast differentiation [43,44,45,46,47]. Furthermore, five flavonoids, namely isoquercitrin, kaempferitrin, epicatechin, quercitrin, and naringenin, have been shown to have anti-osteoporotic effects [48,49,50,51,52,53,54,55,56]. Isoquercitrin has shown anti-osteoporotic activity in ovariectomized rats by inhibiting hypoxia-inducible factor-1 alpha [48], while kaempferitrin exerts protective effects against OVX-related bone loss and microarchitecture deterioration [49]. Quercitrin has been reported to inhibit osteoclast differentiation in RAW264.7 cells, stimulate osteoblast differentiation in MC3T3-E1 cells in vitro, and attenuate osteoporosis in ovariectomized rats by increasing the expression of alkaline phosphatase [50,51,52]. Additionally, studies have shown that epicatechin and naringenin inhibit osteoclast differentiation and prevent OVX-induced bone loss [53,54,55,56]. Therefore, the presence of these phytochemical constituents in EEEA suggests potential biological efficacy in exerting anti-osteoclastic and anti-osteoporotic effects through the synergistic actions among various phytochemicals.

In summary, our study has unveiled the previously unexplored potential of EEEA in conferring bone protection. Its ability to inhibit osteoclast differentiation and function, combined with the presence of phytochemical constituents possessing bone-protective properties, along with its demonstrated effectiveness in OVX mice, collectively position it as a promising candidate for addressing a range of pathological bone loss conditions marked by excessive osteoclast activity. However, further investigations are essential to elucidate various aspects, including in vivo efficacy, safety profile, and the composition of active constituents within EEEA. These inquiries are crucial for its prospective development as a therapeutic agent against a spectrum of pathological bone diseases.

## 4. Conclusions

In conclusion, the findings of our study suggest that EEEA holds promising potential as a therapeutic agent for the prevention and treatment of osteoporosis. EEEA effectively inhibited osteoclastogenesis by indirectly reducing RANKL expression in osteoclast-supporting cells and directly inhibiting RANKL-mediated signaling pathways for osteoclastogenesis. In addition, EEEA exhibited anti-resorptive properties in mature osteoclasts. Moreover, in an OVX-induced mouse model of osteoporosis, EEEA administration significantly mitigated bone loss, indicating its potential preventive effect in postmenopausal osteoporosis. Collectively, these findings highlight EEEA as a potential therapeutic agent with anti-osteoporotic effects, presenting new possibilities for osteoporosis management. Further research and clinical studies are warranted to validate the efficacy and safety of EEEA as a treatment for osteoporosis.

## Figures and Tables

**Table 1:** The phytochemical profiles of EEEA were analyzed using UHPLC-MS/MS.

No	Retention Time (min)	Ion Mode	Error (ppm)	Formula	Expected Mass (*m*/*z*)	Measured Mass (*m*/*z*)	MS/MS Fragments (*m*/*z*)	Identifications
1	5.09	${\left[M-H\right]}^{-}$	1.8547	C15H14O6	289.0718	289.0723	289.0722, 245.0819, 109.028	Catechin *
2	5.60	${\left[M-H\right]}^{-}$	2.1714	C15H14O6	289.0718	289.0724	289.0721, 245.0818, 203.0709, 125.0323	Epicatechin *
3	5.83	${\left[M-H\right]}^{-}$	2.5901	C27H30O17	625.141	625.1426	439.6026, 421.9533, 300.0278, 151.0027	Quercetin 3-O-sophoroside *
4	6.11	${\left[M-H\right]}^{-}$	4.266	C26H28O16	595.136	595.1319	300.0277, 271.0239, 224.6036, 151.0025	Quercetin 3-O-sambubioside
5	6.72	${\left[M-H\right]}^{-}$	2.561	C21H20O12	463.0882	463.0894	303.0411, 300.0278, 299.0195, 272.0279	Isoquercitrin
6	6.82	${\left[M-H\right]}^{-}$	1.5343	C27H30O14	577.1563	577.1572	431.1940, 285.0475	Kaempferitrin *
7	7.47	${\left[M-H\right]}^{-}$	2.4164	C21H20O11	447.0933	447.0944	301.0347, 300.0269	Quercitrin *
8	8.09	${\left[M-H\right]}^{-}$	1.6359	C15H12O6	287.0561	287.0566	287.0563, 259.0612, 243.0661, 125.023	Dihydrokaempferol *
9	10.73	${\left[M-H\right]}^{-}$	1.6436	C15H12O5	271.0612	271.0616	271.0614, 151.0025, 119.0489, 93.0331	Naringenin *
10	20.57	${\left[M-H\right]}^{-}$	2.386	C30H48O3	455.3531	455.3542	437.3282, 327.2552, 281.2494	Betulinic acid *
11	20.79	${\left[M-H\right]}^{-}$	2.6541	C30H48O3	455.3531	455.3543	455.3535, 437.3281, 393.3023	Oleanolic acid *

*, compared with the retention time and MS spectral data of an authentic standards.

## Data Availability

All data of this study are provided within this study.

## References

[1] Sims N.A., Gooi J.H. (2008). Bone remodeling: Multiple cellular interactions required for coupling of bone formation and resorption. Semin. Cell. Dev. Biol..

[2] Zhao X., Patil S., Xu F., Lin X., Qian A. (2021). Role of Biomolecules in Osteoclasts and Their Therapeutic Potential for Osteoporosis. Biomolecules.

[3] Hak D.J. (2018). The biology of fracture healing in osteoporosis and in the presence of anti-osteoporotic drugs. Injury.

[4] Mbese Z., Aderibigbe B.A. (2021). Bisphosphonate-Based Conjugates and Derivatives as Potential Therapeutic Agents in Osteoporosis, Bone Cancer and Metastatic Bone Cancer. Int. J. Mol. Sci..

[5] Nardone V., D’Asta F., Brandi M.L. (2014). Pharmacological management of osteogenesis. Clinics.

[6] Tella S.H., Gallagher J.C. (2014). Biological agents in management of osteoporosis. Eur. J. Clin. Pharmacol..

[7] Chen L.R., Ko N.Y., Chen K.H. (2019). Medical Treatment for Osteoporosis: From Molecular to Clinical Opinions. Int. J. Mol. Sci..

[8] Zhao H., Zhao N., Zheng P., Xu X., Liu M., Luo D., Xu H., Ju D. (2018). Prevention and Treatment of Osteoporosis Using Chinese Medicinal Plants: Special Emphasis on Mechanisms of Immune Modulation. J. Immunol. Res..

[9] Lei S.S., Su J., Zhang Y., Huang X.W., Wang X.P., Huang M.C., Li B., Shou D. (2021). Benefits and mechanisms of polysaccharides from Chinese medicinal herbs for anti-osteoporosis therapy: A review. Int. J. Biol. Macromol..

[10] Kim J.H., Kim N. (2016). Signaling Pathways in Osteoclast Differentiation. Chonnam. Med. J..

[11] Lee K., Seo I., Choi M.H., Jeong D. (2018). Roles of Mitogen-Activated Protein Kinases in Osteoclast Biology. Int. J. Mol. Sci..

[12] Kodama J., Kaito T. (2020). Osteoclast Multinucleation: Review of Current Literature. Int. J. Mol. Sci..

[13] Boyle W.J., Simonet W.S., Lacey D.L. (2003). Osteoclast differentiation and activation. Nature.

[14] Jeong S.Y., Nguyen P.H., Zhao B.T., Ali M.Y., Choi J.S., Min B.S., Woo M.H. (2015). Chemical Constituents of *Euonymus alatus* (Thunb.) Sieb. and Their PTP1B and alpha-Glucosidase Inhibitory Activities. Phytother. Res..

[15] Lee S., Lee D., Baek S.C., Jo M.S., Kang K.S., Kim K.H. (2019). (3beta,16alpha)-3,16-Dihydroxypregn-5-en-20-one from the Twigs of *Euonymus alatus* (Thunb.) Sieb. Exerts Anti-Inflammatory Effects in LPS-Stimulated RAW-264.7 Macrophages. Molecules.

[16] Oh B.K., Mun J., Seo H.W., Ryu S.Y., Kim Y.S., Lee B.H., Oh K.S. (2011). *Euonymus alatus* extract attenuates LPS-induced NF-kappaB activation via IKKbeta inhibition in RAW 264.7 cells. J. Ethnopharmacol..

[17] Jeong E.J., Yang H., Kim S.H., Kang S.Y., Sung S.H., Kim Y.C. (2011). Inhibitory constituents of *Euonymus alatus* leaves and twigs on nitric oxide production in BV2 microglia cells. Food Chem. Toxicol..

[18] Lee S., Moon E., Choi S.U., Kim K.H. (2016). Lignans from the Twigs of *Euonymus alatus* (Thunb.) Siebold and Their Biological Evaluation. Chem. Biodivers.

[19] Fan L., Zhang C., Ai L., Wang L., Li L., Fan W., Li R., He L., Wu C., Huang Y. (2020). Traditional uses, botany, phytochemistry, pharmacology, separation and analysis technologies of *Euonymus alatus* (Thunb.) Siebold: A comprehensive review. J. Ethnopharmacol..

[20] Wang Z.L., Sun H.H., Liu H.Y., Ji Q.X., Niu Y.T., Ma P., Hao G., Zhang J.X., Yuan Y.Y., Chai X.L. (2022). The water extracts of *Euonymus alatus* (Thunb.) Siebold attenuate diabetic retinopathy by mediating angiogenesis. J. Ethnopharmacol..

[21] Gu D.R., Yang H., Kim S.C., Hwang Y.H., Ha H. (2022). Water Extract of Piper longum Linn Ameliorates Ovariectomy-Induced Bone Loss by Inhibiting Osteoclast Differentiation. Nutrients.

[22] Woo Y., Lim J.S., Oh J., Lee J.S., Kim J.S. (2020). Neuroprotective Effects of *Euonymus alatus* Extract on Scopolamine-Induced Memory Deficits in Mice. Antioxidants.

[23] Gurung P., Shrestha R., Lim J., Thapa Magar T.B., Kim H.H., Kim Y.W. (2022). *Euonymus alatus* Twig Extract Protects against Scopolamine-Induced Changes in Brain and Brain-Derived Cells via Cholinergic and BDNF Pathways. Nutrients.

[24] Shin D.Y., Kim B.S., Lee H.Y., Park Y.M., Kim Y.W., Kim M.J., Yang H.J., Kim M.S., Bae J.S. (2023). *Euonymus alatus* (Thunb.) Siebold leaf extract enhanced immunostimulatory effects in a cyclophosphamide-induced immunosuppressed rat model. Food Nutr. Res..

[25] Fu Q., Wang H., Lan Y., Li S., Hashi Y., Chen S. (2014). High-throughput and sensitive screening of compounds with deoxyribonucleic acid-binding activity by a high-performance liquid chromatography-tandem mass spectrometry-fluorescence detection technique using palmatine as a fluorescence probe. J. Chromatogr. A.

[26] Liu X., Wang Y., Ge W., Cai G., Guo Y., Gong J. (2022). Spectrum-effect relationship between ultra-high-performance liquid chromatography fingerprints and antioxidant activities of Lophatherum gracile Brongn. Food Sci. Nutr..

[27] Zhai X., Lenon G.B., Xue C.C., Li C.G. (2016). *Euonymus alatus*: A Review on Its Phytochemistry and Antidiabetic Activity. Evid. Based Complement. Altern. Med..

[28] Ghiulai R., Mioc M., Racoviceanu R., Prodea A., Milan A., Coricovac D., Dehelean C., Avram S., Zamfir A.D., Munteanu C.V.A. (2022). Structural Investigation of Betulinic Acid Plasma Metabolites by Tandem Mass Spectrometry. Molecules.

[29] Choi C.-I., Lee S.R., Kim K.H. (2015). Antioxidant and α-glucosidase inhibitory activities of constituents from *Euonymus alatus* twigs. Ind. Crops Prod..

[30] Kang H.R., Eom H.J., Lee S.R., Choi S.U., Kang K.S., Lee K.R., Kim K.H. (2015). Bioassay-guided Isolation of Antiproliferative Triterpenoids from *Euonymus alatus* Twigs. Nat. Prod. Commun..

[31] Tobeiha M., Moghadasian M.H., Amin N., Jafarnejad S. (2020). RANKL/RANK/OPG Pathway: A Mechanism Involved in Exercise-Induced Bone Remodeling. BioMed Res. Int..

[32] Honma M., Ikebuchi Y., Kariya Y., Suzuki H. (2014). Regulatory mechanisms of RANKL presentation to osteoclast precursors. Curr. Osteoporos. Rep..

[33] Boyce B.F., Rosenberg E., de Papp A.E., Duong L.T. (2012). The osteoclast, bone remodelling and treatment of metabolic bone disease. Eur. J. Clin. Investig..

[34] Boyce B.F., Xing L. (2008). Functions of RANKL/RANK/OPG in bone modeling and remodeling. Arch. Biochem. Biophys..

[35] Park J.H., Lee N.K., Lee S.Y. (2017). Current Understanding of RANK Signaling in Osteoclast Differentiation and Maturation. Mol. Cells.

[36] Nakashima T., Takayanagi H. (2011). New regulation mechanisms of osteoclast differentiation. Ann. N. Y. Acad. Sci..

[37] Kim J.H., Kim N. (2014). Regulation of NFATc1 in Osteoclast Differentiation. J. Bone Metab..

[38] Amarasekara D.S., Yun H., Kim S., Lee N., Kim H., Rho J. (2018). Regulation of Osteoclast Differentiation by Cytokine Networks. Immune Netw..

[39] Izawa T., Arakaki R., Mori H., Tsunematsu T., Kudo Y., Tanaka E., Ishimaru N. (2016). The Nuclear Receptor AhR Controls Bone Homeostasis by Regulating Osteoclast Differentiation via the RANK/c-Fos Signaling Axis. J. Immunol..

[40] Izawa T., Arakaki R., Ishimaru N. (2017). Crosstalk between Cytokine RANKL and AhR Signalling in Osteoclasts Controls Bone Homeostasis. J. Cytokine Biol..

[41] Khosla S., Oursler M.J., Monroe D.G. (2012). Estrogen and the skeleton. Trends Endocrinol. Metab..

[42] Weitzmann M.N., Pacifici R. (2006). Estrogen deficiency and bone loss: An inflammatory tale. J. Clin. Investig..

[43] Wei J., Li Y., Liu Q., Lan Y., Wei C., Tian K., Wu L., Lin C., Xu J., Zhao J. (2020). Betulinic Acid Protects From Bone Loss in Ovariectomized Mice and Suppresses RANKL-Associated Osteoclastogenesis by Inhibiting the MAPK and NFATc1 Pathways. Front. Pharmacol..

[44] Jeong D.H., Kwak S.C., Lee M.S., Yoon K.H., Kim J.Y., Lee C.H. (2020). Betulinic Acid Inhibits RANKL-Induced Osteoclastogenesis via Attenuating Akt, NF-kappaB, and PLCgamma2-Ca(2+) Signaling and Prevents Inflammatory Bone Loss. J. Nat. Prod..

[45] Zhao D., Li X., Zhao Y., Qiao P., Tang D., Chen Y., Xue C., Li C., Liu S., Wang J. (2018). Oleanolic acid exerts bone protective effects in ovariectomized mice by inhibiting osteoclastogenesis. J. Pharmacol. Sci..

[46] Zhao D., Shu B., Wang C., Zhao Y., Cheng W., Sha N., Li C., Wang Q., Lu S., Wang Y. (2020). Oleanolic acid exerts inhibitory effects on the late stage of osteoclastogenesis and prevents bone loss in osteoprotegerin knockout mice. J. Cell. Biochem..

[47] Xie B.P., Shi L.Y., Li J.P., Zeng Y., Liu W., Tang S.Y., Jia L.J., Zhang J., Gan G.X. (2019). Oleanolic acid inhibits RANKL-induced osteoclastogenesis via ER alpha/miR-503/RANK signaling pathway in RAW264.7 cells. Biomed. Pharmacother..

[48] Fayed H.A., Barakat B.M., Elshaer S.S., Abdel-Naim A.B., Menze E.T. (2019). Antiosteoporotic activities of isoquercitrin in ovariectomized rats: Role of inhibiting hypoxia inducible factor-1 alpha. Eur. J. Pharmacol..

[49] Ma X.Q., Han T., Zhang X., Wu J.Z., Rahman K., Qin L.P., Zheng C.J. (2015). Kaempferitrin prevents bone lost in ovariectomized rats. Phytomedicine.

[50] Xing L.Z., Ni H.J., Wang Y.L. (2017). Quercitrin attenuates osteoporosis in ovariectomized rats by regulating mitogen-activated protein kinase (MAPK) signaling pathways. Biomed. Pharmacother..

[51] Satue M., Arriero Mdel M., Monjo M., Ramis J.M. (2013). Quercitrin and taxifolin stimulate osteoblast differentiation in MC3T3-E1 cells and inhibit osteoclastogenesis in RAW 264.7 cells. Biochem. Pharmacol..

[52] Choi E.M. (2012). Protective effect of quercitrin against hydrogen peroxide-induced dysfunction in osteoblastic MC3T3-E1 cells. Exp. Toxicol. Pathol..

[53] Lin T.H., Yang R.S., Wang K.C., Lu D.H., Liou H.C., Ma Y., Chang S.H., Fu W.M. (2013). Ethanol Extracts of Fresh Davallia formosana (WL1101) Inhibit Osteoclast Differentiation by Suppressing RANKL-Induced Nuclear Factor- kappa B Activation. Evid. Based Complement. Altern. Med..

[54] Wang W., Wu C., Tian B., Liu X., Zhai Z., Qu X., Jiang C., Ouyang Z., Mao Y., Tang T. (2014). The inhibition of RANKL-induced osteoclastogenesis through the suppression of p38 signaling pathway by naringenin and attenuation of titanium-particle-induced osteolysis. Int. J. Mol. Sci..

[55] La V.D., Tanabe S., Grenier D. (2009). Naringenin inhibits human osteoclastogenesis and osteoclastic bone resorption. J. Periodontal. Res..

[56] Wang W., Li M., Luo M., Shen M., Xu C., Xu G., Chen Y., Xia L. (2018). Naringenin inhibits osteoclastogenesis through modulation of helper T cells-secreted IL-4. J. Cell. Biochem..

